# Exemestane after tamoxifen as adjuvant hormonal therapy in postmenopausal women with breast cancer: effects on body composition and lipids

**DOI:** 10.1038/sj.bjc.6603258

**Published:** 2006-07-11

**Authors:** G Francini, R Petrioli, A Montagnani, A Cadirni, S Campagna, E Francini, S Gonnelli

**Affiliations:** 1 Department of Human Pathology and Oncology, Medical Oncology Section, University of Siena, Siena, Italy; 2 Department of Metabolic and Endocrinological Science and Biochemistry, University of Siena, Siena, Italy

**Keywords:** body weight, exemestane, hormonal therapy, lipid, tamoxifen

## Abstract

Recent studies have shown that administering the aromatase inhibitor exemestane after 2–3 years of tamoxifen therapy significantly improves disease-free survival in postmenopausal women with primary breast cancer in comparison with standard 5-year tamoxifen treatment. Although many of the adverse effects associated with exemestane and tamoxifen have been analysed, there are no comparative data concerning body weight and body composition. The aim of this randomised study was to evaluate the longitudinal changes in body composition and lipid profiles in postmenopausal women switched from tamoxifen to exemestane. In total, 60 overweight or obese postmenopausal patients were enrolled. Their anthropometric data, body composition, including fat mass (FM) and fat-free mass (FFM), and lipid profiles, caloric intake and physical activity were assessed 1 week before randomisation, and 6 and 12 months later. In all, 55 patients (27 on tamoxifen and 28 on exemestane) completed the 1-year study period. Fat mass had significantly decreased by month 12 in the exemestane, but not in the tamoxifen group; the between-group difference was statistically significant (*P*<0.01). The FFM/FM ratio had significantly increased in the exemestane group, but not the tamoxifen group; the between-group difference was statistically significant (*P*<0.05). Triglycerides and high-density lipoprotein cholesterol significantly decreased (*P*<0.01; *P*<0.05), and low-density lipoprotein cholesterol significantly increased (*P*<0.01) in the exemestane group at the end of the 1-year study period. Our findings suggest that switching patients to adjuvant exemestane treatment after at least 2 years of tamoxifen therapy may be associated with an advantage over continuing adjuvant tamoxifen treatment in terms of body composition.

Tamoxifen is currently used to treat all stages of oestrogen receptor-positive breast cancer and, in the adjuvant setting, has substantial benefits in terms of disease-free and overall survival (Early Breast Cancer Trialists' Collaborative Group, 1992).

The major symptoms attributable to tamoxifen therapy are hot flushes, sweating and vaginal discharges. Although serious adverse medical effects are rare, women are often concerned about some of the risks of tamoxifen therapy, such as blood clots, strokes and endometrial cancer (Meier and Jick, 1998; Ganz, 2001). Moreover, they have often reported a range of events attributed to tamoxifen therapy, including weight gain (Hoskin *et al*, 1992; Rose *et al*, 1993). A checklist-based study of patients on endocrine therapy found that weight gain was a common problem in patients on tamoxifen (Malinovszky *et al*, 2004), but the results of large-scale, randomised clinical trials such as NSABP-B14 and NSABP-P1 suggest that adjuvant tamoxifen therapy is not associated with an increased risk of weight gain, although it must be pointed out that they considered only body weight and not body composition (Fisher *et al*, 1989, 1998). Nevertheless, adult weight gain is largely reflected in increased body fat, which may be more suitable for assessing adiposity and its metabolic consequences than body weight or the body mass index (BMI), which reflect both lean and fat body mass (Abu-Abid *et al*, 2002).

The negative effects of excess body weight and fat mass (FM) on the recurrence of breast cancer, and the survival of both pre- and postmenopausal women, has recently been underlined (Daling *et al*, 2001).

Third-generation aromatase inhibitors, which are considered a new development in the endocrine treatment of oestrogen receptor-positive breast cancer in postmenopausal women, prevent the conversion of adrenal androgens into oestrogens by inhibiting the aromatase of the cytochrome P450-dependent enzyme that is responsible for the majority of oestrogen production in postmenopausal women as well as in men (Smith and Dowsett, 2003).

One of the three newest aromatase inhibitors is exemestane, whose irreversible binding to the aromatase enzyme causes permanent inactivation even after the drug is cleared from the circulation (Goss and Strasser, 2001). Recent findings have shown that, in comparison with standard 5-year tamoxifen therapy, the administration of exemestane after 2–3 years of tamoxifen significantly improves disease-free survival in postmenopausal women with primary breast cancer (Coombes *et al*, 2004). Although many of the adverse effects associated with tamoxifen and exemestane have been analysed, there are no comparative data concerning their effects on body composition and body weight; furthermore, there are few published data concerning the impact of adjuvant exemestane therapy on lipid profiles (Atalay *et al*, 2004; Markopoulos *et al*, 2005). The aim of this study was to evaluate the changes in the body composition and lipid profiles of postmenopausal women switched from tamoxifen to exemestane.

## PATIENTS AND METHODS

### Eligibility criteria

The study was approved by our Institutional Review Board, and all of the patients gave their written informed consent.

The eligible patients were postmenopausal oestrogen receptor-positive women with resected breast cancer aged less than 75 years, who had a BMI of 25–35 (weight(kg)/height(m)^2^) and had received at least 2 years' adjuvant tamoxifen treatment. Postmenopausal status was defined as an age of 55 years or more with amenorrhea for more than 2 years, or amenorrhea for more than 1 year at the time of diagnosis. The exclusion criteria were any evidence of a local relapse or distant metastasis, clinical signs of severe osteoporosis, the use of medications that could affect lipid levels, or the presence of serious medical conditions that could alter food absorption or prognosis. The subjects were randomised using a computer-generated sequence; the person responsible for the randomisation and the people making the study measurements were blinded. The study was not nested in a larger randomised trial.

In total, 60 patients met the eligibility criteria and were randomised to continue tamoxifen 20 mg day^−1^ (*n*=30) or to switch to exemestane 25 mg day^−1^ (*n*=30). The two groups were well balanced in terms of their main baseline characteristics, including height, weight and BMI (Table 1). All of the enrolled women were overweight or obese.

### Measurements

The patients' anthropometric data (including height, weight and BMI), body composition, lipid profiles, physical activity, food intake and quality of life (QOL) were assessed 1 week before randomisation, and after 6 and 12 months of treatment. Weight was measured using a balance beam scale after an overnight fast.

### Lipid metabolism

Fasting venous blood samples were drawn at all visits in order to assess the levels of total cholesterol (TC), triglycerides (TG) and high- and low-density lipoprotein cholesterol (HDL-C and LDL-C), which except for LDL-C (calculated using Friedwald's formula) were measured by means of colorimetry (Autoanalyzer, Menarini, Italy). The intra- and interassay coefficients of variation in our Institution are, respectively, 1.8 and 3.9% for TC, 1.7 and 3.0% for TG, and 2.0 and 3.0% for HDL-C.

All of the individual samples were stored at −80°C until analysis when they were assayed using one batch of reagent.

### Body composition

Body composition was evaluated by means of a single, whole-body, dual-energy X-ray absorptiometry scan made using a QDR 4500 Hologic machine (DXA Hologic Inc., Waltham, MA, USA), and the results were used to derive FM, and fat-free mass (FFM).

The DXA measurements were of high quality. Although the method has good long-term stability and precision, consistent performance was further ensured by means of a daily quality control scan using a known standard (the manufacturer's phantom).

### QOL assessment

In order to assess their health-related QOL, all of the patients were asked to complete the European Organisation for Research and Treatment of Cancer (EORTC) core questionnaire (EORTC/QLQ-C30) at trial entry, and after 6 and 12 months (Aaronson *et al*, 1993). The questionnaire consists of 30 items covering general physical symptoms and function, fatigue/malaise, and social and emotional functioning: higher function scores indicate better functioning, whereas higher symptom scores indicate worse symptoms.

During the QOL interview, the women provided information concerning exercise (recorded as the number of hours/week devoted to physical activity) and food intake, which was measured by means of a food frequency questionnaire translated into Italian (Sallis *et al*, 1985; Block *et al*, 1986).

### Statistical analysis

Assuming an increase of about 15% in serum LDL-C in women switched from tamoxifen to exemestane, a randomised design required 30 patients per group to verify a statistical significant difference in this parameter with an *α* of 0.05 and a *β* of 0.80.

Analysis of variance for paired data was used to evaluate the within-group variations in all of the parameters, and analysis of variance for unpaired data to evaluate between-group differences after having calculated the percentage changes at each visit. Nonparametric tests were used as appropriate. A *P*-value of <0.05 was considered statistically significant for all of the tests, which were made using SPSS 10.1 statistical software.

## RESULTS

Of the 60 enrolled patients, 55 (27 on tamoxifen and 28 on exemestane) completed the 1-year study period, three were lost to follow-up, and two had concomitant diseases unrelated to the study drugs (not cardiovascular complications). Table 1 shows the characteristics of the 55 study completers included in the analysis.

Table 2 shows the mean (±s.d.) weight, caloric intake and physical activity in the two groups at baseline, and after 6 and 12 months. Weight remained unchanged in the tamoxifen group (69.75±6.21 kg at baseline and 69.17±7.33 kg at month 12), but decreased in the exemestane group (69.88±7.58 kg at baseline and 67.89±7.45 kg at month 12; *P*=0.06). The questionnaire data showed no statistically significant differences in baseline caloric intake or physical activity between the tamoxifen and exemestane group, and no statistically significant changes were observed in either group after six and 12 months.

**Figure 1:**
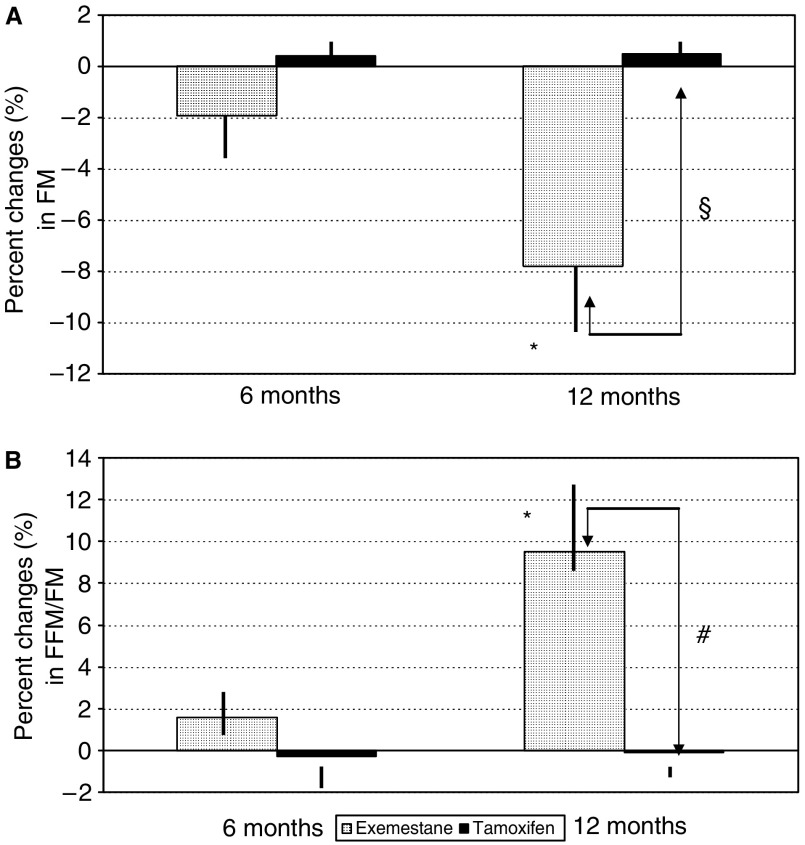
Percentage changes in FM (**A**) and the FFM/FM ratio (**B**) in the exemestane and tamoxifen groups after 6 and 12 months (^*^P<0.01 *vs* baseline; #*P*<0.05, §between groups).

Fat mass had significantly decreased by month 12 in the exemestane group (*P*<0.01) but not in the tamoxifen group; the between-group difference was statistically significant (*P*<0.01) (Figure 1A): The FFM/FM ratio significantly increased by month 12 in the exemestane group (*P*<0.01) but not in the tamoxifen group; the between-group difference was statistically significant (*P*<0.05) (Figure 1B).

There were no statistically significant changes in the lipid profiles of the patients treated with tamoxifen (Table 3). In the exemestane group, HDL-C decreased by 9.1% at month 6 and by 12.0% at month 12 when the difference became statistically significant (*P*<0.05), LDL-C significantly increased by 14.8% at month 6 and 16.5% at month 12 (*P*<0.01), and TG decreased by 12.1% at month 6 and 16.9% at month 12 (*P*<0.01). The between-group differences were statistically significant only for LDL-C (*P*<0.05).

The comparison of the linearly transformed mean values of the EORTC-QLQ-C30 data was performed: the very similar global health status (*P*=0.9) and global QOL scores (*P*=0.9) of the two groups indicated that there were no statistically significant differences in their baseline QOL. In comparison with baseline, none of the *P-*values in the functional and symptom scales showed any clear between-group trend at month 6 but, at month 12, there was a trend toward improved physical functioning, global health status and global QOL scores in the exemestane group; the between-group difference was not statistically significant.

## DISCUSSION

Tamoxifen has long been the gold standard therapy for postmenopausal women with hormone receptor-positive breast cancer but, although the drug is generally well tolerated, its prolonged use may be associated with various side effects, including gynecological and thromboembolic complications (Fisher *et al*, 1998). Moreover, a few studies have reported some changes in body weight (especially weight gains) in women with resected breast cancer receiving adjuvant treatment including tamoxifen (Camoriano *et al*, 1990; Chlebowski *et al*, 2002; Malinovszky *et al*, 2004).

The present study suggests, for the first time, that switching overweight or obese patients to adjuvant treatment with exemestane after at least 2 years of tamoxifen therapy is associated with a significant decrease in FM (Figure 1), with no statistical significant difference in body weight (Table 2). It must be remembered that the precise pathophysiological mechanisms of body weight changes remain unclear in many patients and that, as body weight is determined by an interplay of caloric intake, activity level and metabolic rate, significant alterations in any of these factors may lead to weight loss or gain (Levine *et al*, 1991; Goodwin *et al*, 1998). However, we observed no statistically significant differences in caloric intake or physical activity between our two randomised groups during the study period (Table 2).

One possible explanation of our findings is that the pharmacological properties of exemestane are different from those of tamoxifen. The effects of tamoxifen on the human body vary and can be characterised as a mixture of oestrogenic and anti-oestrogenic properties, whereas exemestane inhibits *in vivo* aromatisation by about 98% and has no partial agonist activity, thus leading to a profound decrease in serum oestrogen levels (Furr and Jordan, 1984; Geisler *et al*, 1998). Two-thirds of breast tumours are oestrogen-dependent, and aromatase inhibitors can help block the growth of these tumours by lowering the amount of oestrogen in the body (Yeu and Santen, 1996). As it is known that exemestane and the other aromatase inhibitors reduce circulating oestrogen levels, and that oestrogens have direct effects on adipocytes and the other cellular constituents of adipose tissue, there may be an association between exemestane use, reduced circulating oestrogen levels and body weight changes (Siiteri, 1987; Selby, 1990; Hankinson *et al*, 1995). However, if such an association exists, our data do not offer any evidence as to whether the expected decrease in oestrogen levels was the cause or effect of body weight changes.

The use of exemestane may also influence appetite, but our QoL questionnaire did not reveal any statistically significant changes in appetite in our study population.

Another intriguing point is the effect of tamoxifen and exemestane on the lipid profiles of postmenopausal women. It has long been known that oestrogens lower serum cholesterol, and that the agonist activity of the selective oestrogen-receptor modulator tamoxifen has protective effects on lipid profiles (Love *et al*, 1990). Nevertheless, it has recently been suggested that women receiving aromatase inhibitors for breast cancer prevention may be at increased risk of cardiovascular diseases, because the oestrogen-lowering effects of such drugs may have adverse effects on blood lipids (Baum *et al*, 2003; Goss *et al*, 2003; Atalay *et al*, 2004; Esteva and Hortobagyi, 2006; Markopoulos *et al*, 2005). Our data showed a significant increase in LDL-C in the patients treated with exemestane: this finding may seem to differ from those of other studies indicating that exemestane has a neutral effect on LDL-C, but it must be remembered that our patients had been treated with tamoxifen for at least 2 years, which markedly reduces LDL-C levels (Love *et al*, 1990; Atalay *et al*, 2004; Esteva and Hortobagyi, 2006; Markopoulos *et al*, 2005). The increase in LDL-C may therefore be at least partially explained by the loss of the positive action of tamoxifen. The decrease in HDL-C in the exemestane group is in line with previous studies, and may be explained by a direct action of exemestane and/or by the interruption of tamoxifen therapy (Atalay *et al*, 2004; Lonning *et al*, 2005; Markopoulos *et al*, 2005; Esteva and Hortobagyi, 2006).

The Intergroup Exemestane Study found a trend toward more frequent myocardial infarction in patients treated with exemestane than in those treated with tamoxifen (Coombes *et al*, 2004). We found that 1-year's use of exemestane led to more changes in cholesterol parameters, which are commonly associated with an increased risk of coronary heart disease (Table 3), thus indicating that the lipid profiles of patients receiving exemestane or other aromatase inhibitors should be monitored. However, it must be borne in mind that the genesis of cardiovascular diseases is multifactorial, and many authors have found that exemestane treatment significantly reduces triglyceride levels, which may help to balance the negative effect of the decrease in HDL-C (Atalay *et al*, 2004; Markopoulos *et al*, 2005). Only large-scale randomised trials and a long follow-up can answer the question as to whether postmenopausal women treated with exemestane or other aromatase inhibitors for 3–5 years are at greater cardiovascular risk than those treated with tamoxifen.

In conclusion, the results of our study suggest that adjuvant exemestane treatment may have an advantage over adjuvant tamoxifen treatment in terms of body composition. In addition to recent findings that the use of exemestane after 2–3 years of tamoxifen therapy significantly improves disease-free survival in comparison with standard 5-year tamoxifen treatment, this could be a further factor worth considering when deciding on adjuvant hormonal therapy for postmenopausal women with breast cancer.

## Figures and Tables

**Table 1:** Baseline characteristics of the study population

	**Tamoxifen (*n*=27)**	**Exemestane (*n*=28)**
Age (years)	61.15±2.74	61.89±4.45
*Tumour stage*		
T1	46%	38%
T2	36%	40%
T3	18%	22%
		
*Node status*		
N+(1–3)	21%	18%
N+(>4)	8%	15%
Negative	71%	67%
		
*Estrogen receptor status*		
Positive	100%	100%
Negative	0	0
		
*Progesteron receptor status*		
Positive	72%	65%
Negative	28%	35%
		
*Primary treatment*		
Mastectomy	61%	54%
Radiotherapy	42%	51%
Chemotherapy	24%	27%
		
Height, cm (mean±s.d.)	156.4±2.06	155.96±1.92
Weight, kg (mean±s.d.)	69.75±6.21	69.88±7.58
BMI, kg/m^2^ (mean±s.d.)	28.97±1.88	29.17±2.12

BMI=body mass index.

**Table 2:** Weight, caloric intake and physical activity data: mean values (±s.d.) on tamoxifen and exemestane therapy

	**Tamoxifen**					
**Exemestane**	**Baseline**	**6th month**	**12th month**	**Baseline**	**6th month**	**12th month**
Weight (kg)	69.75±6.21 (CI:67.41–72.09)	68.33±7.04 (CI:65.67–70.99)	69.17±7.33 (CI:66.41–71.93)	69.88±7.58 (CI:67.07–72.09)	67.95±6.12 (CI:65.68–70.22)	67.89± 7.45 (CI:65.13–70.65)
Caloric intake (kcal day^−1^)	1832±326 (CI:1709–1955)	1852±351 (CI:1720–1984)	1848±367 (CI:1709–1986)	1841±365 (CI:1705–1976)	1837±326 (CI:1716–1957)	1818±312 (CI:1702–1933)
Physical activity (h week^−1^)	11.81±7.60 (CI:8.94–14.68)	12.12±8.25 (CI:9.01–15.23)	13.22± 8.46 (CI:10.03–16.41)	10.28±7.26 (CI:7.59–12.97)	11.80±8.33 (CI:8.71–14.89)	11.18±7.25 (CI:8.49–13.87)

CI=confidence interval.

**Table 3:** Lipide profile: mean values (±s.d.) on tamoxifen and exemestane therapy

	**Tamoxifen**					
**Exemestane**	**Baseline**	**6th month**	**12th month**	**Baseline**	**6th month**	**12th month**
TC (mg dl^−1^)	215.12±10.01 (CI:211.34–218.90)	216.23±10.44 (CI:212.29–220.17)	215.32±11.21 (CI:211.09–219.55)	215.68±9.31 (CI:212.23–219.13)	224.27±13.33 (CI:219.33–229.21)	224.17±12.83 (CI:219.42–228.92)
HDL-C (mg dl^−1^)	58.62±6.17 (CI:56.29–60.95)	58.22±8.77 (CI:54.91–61.53)	57.98±8.01 (CI:54.96–61.00)	58.07±6.40 (CI:55.7–60.44)	53.41±8.10 (CI:50.41–56.41)	51.20±8.08* (CI:48.21–54.19)
LDL-C (mg dl^−1^)	131.15±14.51 (CI:125–68–136.62)	133.56±19.10 (CI:126.36–140.76)	132.31±19.24 (CI:125.05–139.57)	130.91±15.10 (CI:125.32–136.50)	149.8±17.18* (CI:143.44–156.16)	152.81±18.12**# (CI:146.10–159.52)
TG (mg dl^−1^)	124.87±46.1 (CI:107.48–142.26)	119.30±47.81 (CI:101.27–137.33)	127.63±40.60 (CI:112.32–142.94)	124.11±58.27 (CI:102.53–145.69)	107.21±64.30* (CI:83.39–131.03)	101.04±43.65** (CI:84.87–117.21)

CI=confidence interval; HDL-C=high-density lipoprotein cholesterol; LDL-C=low-density lipoprotein cholesterol; TC=total cholesterol; TG=total triglycerides.

* *P*<0.05,

** *P*<0.01 *vs* baseline values; #*P*<0.05 between groups.
